# Impact of Infection‐Related Immune Responses on ANCA‐Associated Vasculitis in Finns: A Mendelian Randomization Study

**DOI:** 10.1155/genr/3406375

**Published:** 2026-08-23

**Authors:** Naidan Zhang, Caixia Ji, Tong Lv, Xiao Bao

**Affiliations:** ^1^ Department of Laboratory Medicine, Peoples Hospital of Deyang City, Deyang 618000, Sichuan, China, scu.edu.cn; ^2^ Department of Rheumatology, Peoples Hospital of Deyang City, Deyang 618000, Sichuan, China, scu.edu.cn

**Keywords:** ANCA-associated vasculitis, DNA methylation Hannum age acceleration, immune cells, infection, neutrophil extracellular traps, serum-specific antibody

## Abstract

**Background:**

ANCA‐associated vasculitis (AAV) includes microscopic polyarteritis (MPA), granulomatosis with polyangiitis (GPA), and eosinophilic granulomatosis with polyangiitis (EGPA). Studies have revealed that infection‐related immune responses could trigger vasculitis. This study aims to investigate the relationship between infection‐related immune responses and AAV in Finns.

**Methods:**

We used multiple Mendelian randomization (MR) methods and bioinformatics to examine how infection‐related immune responses affect AAV, also investigating the mediation effects of neutrophil extracellular traps (NETs) and aging phenotypes. Bioinformatics data were from the Gene Expression Omnibus (GEO) database. Serum‐specific antibodies were from the U.K. Biobank. AAV subtypes were from FinnGen. Single‐cell sequencing data of immune cells were from the OneK1K database. NETs and aging phenotypes were from the genome‐wide association studies (GWAS).

**Results:**

This study found a preliminary negative correlation between anti‐IgG of HSV1 and the risk of MPA (OR: 0.499, 95% CI: 0.361–0.692, *p* = 3.05E‐05). The SCeQTL analysis revealed that several genes in immune cells were linked to anti‐IgG of HSV1 and MPA, while mediation analysis highlighted DNA methylation Hannum age acceleration (DNAmAA) as a key mediator between gene expression and MPA. HLA‐C expression in CD8nc T cells (*Z* = −2.921, *p* = 0.003, mediation effect = 82.2%) and MDC1 in dendritic cells (*Z* = −3.310, *p* = 0.001, mediation effect = 81.5%) lowered the risk of MPA by downregulating DNAmAA. Conversely, MICA expression in NKR NK cells (*Z* = 3.702, *p* < 0.001, mediation effect = 72.1%) was associated with an increased risk of MPA by upregulating DNAmAA.

**Conclusions:**

This study demonstrated potential correlations between infection‐related immune responses and MPA. The SCeQTL analysis revealed that HLA‐C expression in CD8nc T cells, MDC1 in dendritic cells, and MICA expression in NKR cells were related to the risk of MPA. Furthermore, mediation analysis indicated that DNAmAA exerted a strong mediation effect on the relationship between gene expression and MPA.

## 1. Introduction

Antineutrophil cytoplasmic antibody (ANCA)‐associated vasculitis (AAV) includes microscopic polyarteritis (MPA), granulomatosis with polyangiitis (GPA) and eosinophilic granulomatosis with polyangiitis (EGPA) [1]. Immune responses to infections can trigger vasculitis. For instance, secondary polyarteritis nodosa (PAN) was observed 3 weeks post–anti‐hepatitis B vaccination [2]. Additionally, 10%–54% of patients showed PAN symptoms within six months of HBV infection [3]. Infection‐related immune responses can also lead to vasculitis recurrence. Infection triggers the release of inflammatory mediators, activates endothelial cells and neutrophils, and promotes GPA recurrence [4]. While studies suggest a link between infection and vasculitis, the lack of reliable animal models and the rarity of AAV make it challenging to confirm this relationship through large‐scale clinical studies.

Microorganisms attach to host cells via specific antigens, prompting the host to produce antibodies, such as those against *Mycoplasma pneumoniae*, *Mycobacterium tuberculosis*, and *Treponema pallidum* [5]. Detecting these antibodies is useful for diagnosing infections and evaluating treatment effectiveness. The immune response is linked to the human leukocyte antigen (HLA) [6]. Genome‐wide association studies (GWAS) in Europeans indicate an association between the major histocompatibility complex (MHC) and antimyeloperoxidase (MPO)/antiproteinase 3 (PR3) antigen, with PR3 linked to HLA‐DP and MPO to HLA‐DQ loci at 6p21.32 [7]. These findings suggest that MHC‐related immune response is a risk factor for AAV.

The MHC region, known for polymorphic genes and linkage disequilibrium, complicates statistical analysis. Mendelian randomization (MR) uses genetic variation as instrumental variables (IVs) to assess exposure–outcome relationships, effectively minimizing linkage disequilibrium and weak IVs. This study investigated multiple MR methods and bioinformatics to examine how infection‐related immune responses affect AAV in Finns, also investigating the mediation roles of neutrophil extracellular traps (NETs) and aging phenotypes. These findings aim to enhance understanding of the relationship between infection‐related immune responses and AAV.

## 2. Materials and Methods

### 2.1. Gather Data on Antibodies From Infection‐Related Immune Responses

To collect serum‐specific antibodies from infection‐related immune responses, we used 46 antibody datasets from the U.K. Biobank [8]. These datasets included 13 pathogens from four categories, including herpesviridae, polyomaviridae, bacteria, and parasites (Supporting Table S1). All participants were adults from the United Kingdom. Multiplex serological technology with fluorescent microspheres was used for the detection, measured on the Luminex 100 platform at a 1:1000 dilution. To account for nonspecific antibody interference, we used the UKB‐recommended threshold for significant positive results as the exposure. This threshold had been validated in various infectious agents [9]. We identified single nucleotide polymorphisms (SNPs) with significant differences (*p* < 5 × 10^−8^), no significant linkage disequilibrium (*r* ^2^ < 0.3), and located within ±100 kb of SNPs as IVs.

### 2.2. Gather Data on the AAV Subtypes

We gathered AAV subtypes from FinnGen (Version 11), MPA (166 cases, 451,441 controls), GPA (486 cases, 439,424 controls), and EGPA (118 cases, 439,324 controls). These were the largest AAV subtype datasets (Supporting Table S2). The exposure and outcome were sourced from different European countries to minimize population stratification bias.

### 2.3. Relationship Between Antibodies and AAV

We utilized a two‐sample MR analysis to investigate the relationship between antibodies and AAV, using the inverse variance weighting (IVW) method. Pleiotropy was assessed with the MR‐Egger intercept, heterogeneity was assessed with Cochran’s Q test, and sensitivity was assessed with the leave‐one‐out method. The F‐statistic was used to address weak IV bias. To mitigate the risk of false positive errors from repeated testing on the same dataset, this study utilized the Bonferroni correction to manage the multiple comparison error. The adjusted significance level was calculated by dividing the original *α* value by the number of tests. For instance, the initial significance threshold was set at 0.05, and there were 46 antigen–antibody reaction exposure data; the significance threshold was adjusted to 0.001 (0.05/46), meaning that a *p* value less than 0.001 was deemed statistically significant. Significant SNPs were identified, and the corresponding gene symbols were retrieved from GeneCards. Duplicate values were removed to compile a gene set associated with AAV.

### 2.4. Genes Linked to Immune Response in AAV and Kidney Damage

To investigate the immune response in AAV patients with kidney damage, we analyzed the RNA‐seq dataset GSE108113 from the Gene Expression Omnibus (GEO) database, which included 73 AAV renal samples and 10 healthy controls. This dataset was normalized and analyzed using the “WGCNA” package in R (Version 4.3.1). Genes with intersample differences over 25% were selected, and the “goodSamplesGenes” was applied for hierarchical clustering. The “pickSoftThreshold” was used to estimate a soft threshold with an *R* ^2^ of 0.85. Pearson correlation was used to assess the link between the module and clinical phenotype. The “VennDiagram” package was used to intersect genes. These genes could be crucial in the immune response to infection and renal damage in AAV.

### 2.5. Relationships Between Genes and AAV by SCeQTL

SCeQTL revealed cell type‐specific genes by examining gene expression in various immune cells. We utilized single‐cell RNA sequencing data from the OneK1K database, which contained samples of 1.27 million peripheral blood mononuclear cells (PBMCs) from 982 healthy Europeans. We obtained a dataset of 14 immune cell types with distinct transcriptional profiles, including myeloid cells, monocytes, T cells, and lymphocytes (Supporting Table S3). Data filtering criteria included significant differences (*p* < 1 × 10^−5^), no significant linkage disequilibrium (*r* ^2^ < 0.3), and location within ±100 kb. SCeQTL of immune cell‐specific genes between antibodies and AAV were performed using the “TwoSampleMR” package, and KEGG analysis was used to identify potential enrichment pathways of key genes.

To further substantiate the findings of SCeQTL, we employed Bayesian Weighted Mendelian Randomization (BWMR), a method that incorporated Bayesian statistical principles into the conventional MR. This approach assigned weights based on the quality of SNPs, thereby mitigating the influence of weak instruments and enhancing the robustness of the estimation. Previous research has indicated that utilizing BWMR for cross‐validation was advantageous in bolstering the credibility of the results [10] In interpreting the results, three key aspects were considered: (1) the magnitude and direction of the odds ratio (OR) were consistent with those obtained through IVW; (2) a *p* value less than 0.05 signified a significant causal relationship; and (3) the BWMR results aligned with the IVW method in both direction and significance, indicating a high degree of robustness and resistance to horizontal pleiotropy.

### 2.6. Mediation Analysis of NETs in Immune Response to Infections and AAV

NETs were structures released by neutrophils during immune defense, composed of DNA, histones, and antibacterial proteins like elastase and myeloperoxidase. NETs and related inflammatory indicators were significant risk factors for AAV [11]. The GWAS datasets included NETs, interleukin‐6 (IL‐6), tumor necrosis factor‐alpha (TNF‐a), neutrophil elastase (NE), neutrophil gelatinase‐associated lipocalin (NGAL), MPO, MPO–DNA complexes, and neutrophil‐activating peptide 2 (Supporting Table S4). The data filtering criteria included significant differences (*p* < 1 × 10^−5^), no significant linkage disequilibrium (*r* ^2^ < 0.3), and location within ±100 kb. A two‐sample MR was used to examine the relationship between NETs and AAV. Then, NETs were analyzed as a mediator between serum‐specific antibodies, immune cell‐specific gene expression, and AAV. A mediation effect was confirmed if *p* < 0.0062 (0.05/8), and calculated by a two‐step method.

### 2.7. Mediation Analysis of Aging in Immune Response to Infections and AAV

**FIGURE 1:**
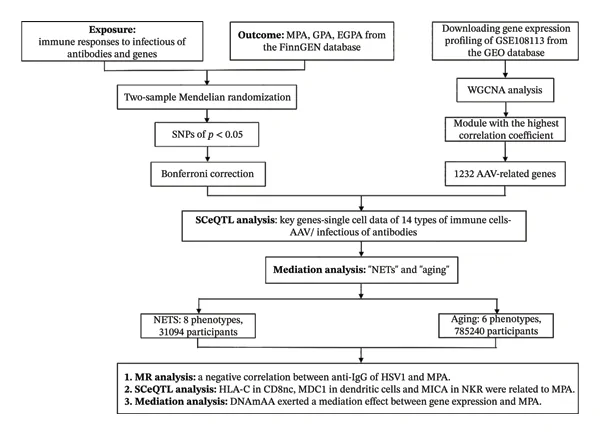
Research flowchart of the study. This study was divided into three parts. The first part conducted a two‐sample MR analysis of infection‐related immune response and AAV. The second part conducted SCeQTL analysis of immune cell‐specific gene expression and AAV. The third part adopted mediation analysis to explore the mediation of NETs and aging. Abbreviations: MPA, microscopic polyarteritis; GPA, granulomatosis with polyangiitis; EGPA, eosinophilic granulomatosis with polyangiitis; GEO, gene expression omnibus; SNP, single nucleotide polymorphism; AAV, antineutrophil cytoplasmic antibody‐associated vasculitis; SCeQTL, single‐cell expression quantitative trait loci. NETs, neutrophil extracellular traps.

Epidemiological studies indicated that AAV rose with age, peaking at 55–74 years in Western Europe and 65–75 years in Northern Europe [12]. We examined the impact of aging on immune response and AAV, focusing on indicators like DNA methylation GrimAge acceleration (DNAmGAA), DNA methylation Hannum age acceleration (DNAmAA), intrinsic epigenetic age acceleration, DNA methylation PhenoAge acceleration, telomere length (TL), and frailty index (FI) (Supporting Table S5). Data filtering and mediation criteria matched those of NETs. All data were sourced from open‐access databases and previous GWAS and required no ethical approval. The research flowchart is shown (Figure 1).

## 3. Results

### 3.1. Relationships Between Antibodies and AAV

In herpesviridae infections, anti‐IgG of herpes simplex virus 1 (HSV1) (OR: 0.499, 95% CI: 0.361–0.692) was negatively correlated with the risk of MPA. Heterogeneity of anti‐IgG and VP1 of Merkel cell polyomavirus was observed (*p*_*Q*_ < 0.05). The results indicated heterogeneity, which precluded their inclusion in subsequent analyses. In the investigation of the association between antibodies and GPA, the presence of heterogeneity was noted, leading to the conclusion that no correlation existed. In the study of the relationship between antibodies and EGPA, no correlation was identified by Bonferroni correction, as detailed in Table 1. Additionally, a preliminary negative correlation between anti‐IgG of HSV1 and the risk of MPA was established using the MR method.

**TABLE 1:** The relationship between the immune response to infection and AAV.

Infectious agents	Exposure	Outcome	*p* value	*p*_*Q*_ value	OR	95% CI
EB	VCA p18	GPA	9.26E‐06	1.77E‐21	2.158	1.536–3.032
EB	ZEBRA	GPA	5.75E‐06	1.21E‐13	1.755	1.376–2.237
HSV1	Anti‐IgG	MPA	3.05E‐05	0.622	0.499	0.361–0.692
MCP	Anti‐IgG	MPA	0.00031	2.69E‐06	0.641	0.503–0.816
MCP	VP1	MPA	0.00021	9.63E‐08	0.369	0.218–0.625
MCP	Anti‐IgG	GPA	4.56E‐06	1.21E‐09	0.689	0.587–0.808

*Note:* AAV, ANCA‐associated vasculitis; MPA, microscopic polyarteritis; GPA, granulomatosis with polyangiitis; EB, Epstein–Barr virus; *p*_*Q*_, *p* value of heterogeneity.

Abbreviations: CI, confidence interval; HSV1, herpes simplex virus 1; MCP, Merkel cell polyomavirus; OR, odds ratio.

### 3.2. Common Genes in Infection‐Related Immune Responses and Renal Damage

**FIGURE 2:**
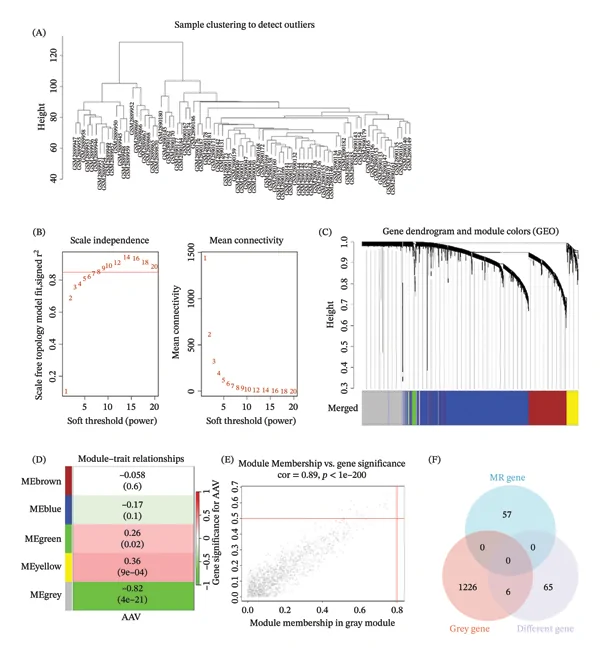
Common genes in infection‐related immune responses and renal damage. (A) Samples were detected in the hierarchical cluster analysis; (B) the adjacency at the optimal power; (C) gene modules were divided and merged using a dynamic shear tree; (D) the grey module had the highest correlation (*r* = 0.82) with renal damage; (E) the correlation coefficient of 1232 genes in the grey module was 0.89; (F) Venn diagrams showed six common genes between the grey module and differentially expressed genes from the GEO database.

Analyzing SNPs with significant differences in the MR analysis, three SNPs linked to AAV were identified (Table 2). Three genes associated with AAV were found through GeneCards (Supporting Table S6). From the GEO database, 22,876 genes were identified between renal damage and controls, with 5719 genes showing over 25% variation. No abnormal samples were detected in the hierarchical cluster analysis (Figure 2A). The optimal power was 8, with an *R* ^2^ of 0.85 (Supporting Table S7). At this power, the adjacency mean approached 0 (Figure 2B). Gene modules were divided and merged using a dynamic shear tree with a correlation coefficient over 0.8 (Figure 2C). Correlation analysis revealed that the grey module had the highest correlation (*r* = 0.82) with renal damage (Figure 2D). A correlation graph was plotted for 1232 genes in the grey module against renal injury, using MM and GS values as axes. The correlation coefficient was 0.89 with a *p* < 0.001, indicating a strong association (Figure 2E). Venn diagrams showed six common genes between the grey module and differentially expressed genes from the GEO database, but no overlap with MR genes, suggesting no shared genes for infection‐related immune response and AAV renal damage (Figure 2F).

**TABLE 2:** Relationship between genes and immune response as well as AAV.

SNP	Antigen targets	Outcome	OR (95% CI)	*p* value
rs199741519	Anti‐IgG	MPA	0.232 (0.087–0.618)	0.003
rs9616098	Anti‐IgG	MPA	0.201 (0.057–0.707)	0.012
rs116029235	Anti‐IgG	MPA	3.449 (1.026–11.593)	0.045

*Note:* AAV, antineutrophil cytoplasmic antibody‐associated vasculitis; MPA, microscopic polyarteritis.

Abbreviations: CI, confidence interval; OR, odds ratio; SNP, single nucleotide polymorphism.

### 3.3. Relationships Between Immune Cell–Specific Genes and Antibodies/AAV

**FIGURE 3:**
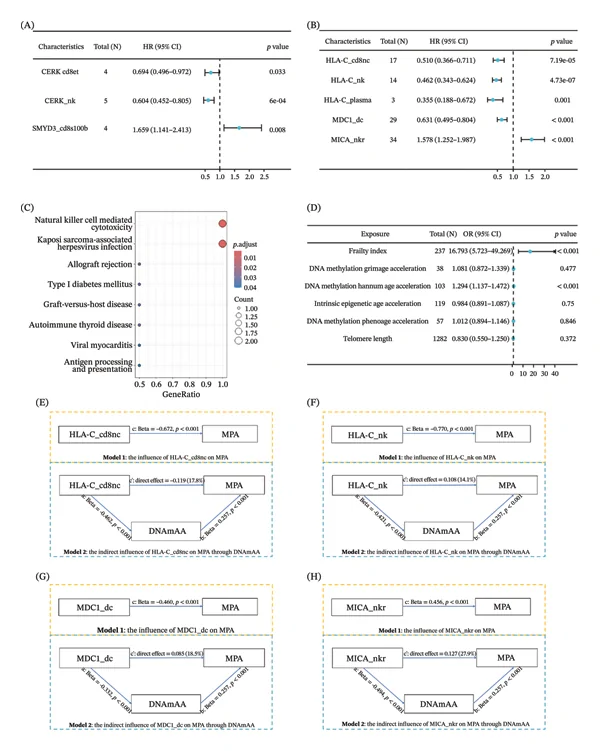
SCeQTL and mediation analysis of aging in immune response to infections and MPA. (A) Expressions of two genes in immune cells affect anti‐IgG of HSV1; (B) expressions of three genes within immune cells might influence MPA; (C) KEGG analysis of above five genes; (D) a positive correlation between both FI and DNAmAA with the risk of MPA; (E) mediation analysis of DNAmAA between HLA‐C_cd8nc and MPA; (F) mediation analysis of DNAmAA between HLA‐C_nk and MPA; (G) mediation analysis of DNAmAA between MDC1_dc and MPA; (H) mediation analysis of DNAmAA between MICA_nkr and MPA. Abbreviations: SCeQTL, single‐cell expression quantitative trait loci; MPA, microscopic polyarteritis; MR, mendelian randomization; DNAmAA, DNA methylation Hannum age acceleration.

The SCeQTL analysis indicated that the expression of two genes within immune cells might affect anti‐IgG of HSV1 through the functional activity of the cells (Figure 3A and Supporting Table S8). Notably, the expression of SMYD3 in cd8s100b cells demonstrated a positive correlation with the production of anti‐IgG (OR: 1.659, *p* = 0.008). Conversely, the expression of CERK in CD8et cells (OR: 0.694, *p* = 0.033) and NK cells (OR: 0.604, *p* = 0.0006) showed negative correlations with anti‐IgG of HSV1. The SCeQTL analysis also revealed that the expression of three genes within immune cells might influence MPA through the activity in these cells (Figure 3B and Supporting Table S9). Specifically, the expression of MHC Class I chain‐associated gene A (MICA) in NKR cells was associated with an increased risk of MPA (OR: 1.578, *p* < 0.001). Conversely, the expression of HLA‐C in CD8nc T cells, NK cells, and plasma immune cells, as well as the expression of MDC1 in dendritic cells, exhibited negative correlations with MPA, thereby reducing the risk (0 < OR < 1). KEGG analysis indicated that these genes were enriched in pathways related to natural killer cell‐mediated cytotoxicity and Kaposi’s sarcoma‐associated herpesvirus infection. These findings suggested that the activation of innate immune cells might play a critical role in modulating the onset and progression of MPA (Figure 3C).

### 3.4. Mediation Analysis of NETs as a Mediator

Initially, we performed the two‐sample MR on NETs and MPA, finding that MPO was associated with an increased risk of MPA (OR: 1.677, *p* < 0.001) (Table 3). No reverse causal relationship was identified (Supporting Table S10). Subsequently, we explored mediation analyses involving “HSV1–MPO–MPA” and “genes within immune cells–MPO–MPA.” The MR analysis revealed no direct causal relationship between anti‐IgG of HSV1 and MPO (*p* > 0.05). Gene expression analysis demonstrated a negative correlation between MICA expression in NKR cells and MPO (OR: 0.841, *p* < 0.001). However, the mediation analysis did not provide evidence supporting a mediating role of MPO in the pathway “genes within immune cells–MPO–MPA” (Supporting Table S11, *p* > 0.05).

**TABLE 3:** Mediation analysis of NETs between antibody/immune cells and MPA.

Exposure	Outcome	NSNP	Beta	OR	95% CI	*p* value
NETs	MPA	28	−0.028	0.973	0.891–1.062	0.535
IL‐6	MPA	12	−0.584	0.558	0.241–1.291	0.173
TNF‐a	MPA	7	0.599	1.820	0.890–3.723	0.101
NE	MPA	39	0.077	1.080	0.870–1.341	0.486
NGAL	MPA	17	0.032	1.033	0.681–1.566	0.879
MPO	MPA	41	0.517	1.677	1.365–2.060	8.17E‐07
MPO–DNA	MPA	12	−0.584	0.558	0.241–1.291	0.173
NAP‐2	MPA	26	0.140	1.150	0.789–1.677	0.467
HSV1/anti‐IgG	MPO	8	−0.019	0.981	−0.129–0.092	0.739
MICA_nkr	MPO	23	−0.173	0.841	0.787–0.899	< 0.001

*Note:* MPA, microscopic polyarteritis; IL‐6, interleukin‐6; MPO, myeloperoxidase; MPO–DNA, myeloperoxidase–DNA complexes.

Abbreviations: CI, confidence interval; NAP‐2, neutrophil‐activating peptide 2; NE, neutrophil elastase; NETs, neutrophil extracellular traps; NGAL, neutrophil gelatinase‐associated lipocalin; OR, odds ratio; TNF‐a, tumor necrosis factor‐alpha.

### 3.5. Mediation Analysis of Aging as a Mediator

A two‐sample MR analysis revealed a positive correlation between both FI and DNAmAA and the risk of MPA (Figure 3D and Supporting Table S12). Notably, FI displayed heterogeneity and pleiotropy, whereas DNAmAA did not exhibit these characteristics (Supporting Table S13). Furthermore, mediation analysis revealed that DNAmAA served as a mediator in the relationship between immune cell gene expression and MPA, with statistical significance (Supporting Table S14). Among them, HLA‐C expression in CD8nc T cells and NK cells was negatively correlated with the risk of MPA by downregulating DNAmAA, with mediation effects of 82.8% and 81.3%, respectively (Figure 3E,F). MDC1 in dendritic cells was negatively correlated with the risk of MPA by downregulating DNAmAA, with a mediation effect of 81.5% (Figure 3G). MICA expression in NKR cells increased the risk of MPA by upregulating DNAmAA, with a mediation effect of 72.1% (Figure 3H).

## 4. Discussion

Patients diagnosed with AAV are susceptible to pulmonary and renal complications. Recognizing these potential risks is essential for informing early therapeutic interventions and enhancing patient prognosis [13]. The study investigated infection‐related immune responses and the risk of AAV in Finns, considering NETs and aging as mediators. A preliminary negative correlation between anti‐IgG of HSV1 and the risk of MPA was established using the MR method. The SCeQTL analysis revealed that HLA‐C expression in CD8nc T cells, MDC1 in dendritic cells, and MICA expression in NKR cells were related to the risk of MPA. Furthermore, mediation analysis indicated that DNAmAA exerted a strong mediation effect between gene expression and MPA. HLA‐C expression in CD8nc T cells and MDC1 in dendritic cells were negatively correlated with the risk of MPA by downregulating DNAmAA. Conversely, MICA expression in NKR cells was associated with the risk of MPA by upregulating DNAmAA. These findings have reference value for clinical use.

The infection‐related immune responses involved three stages (antigen recognition, signal transduction, and immune response execution). In the first stage, antigen‐presenting cells like dendritic cells and macrophages captured and processed pathogens and presented them to T/B cells. During the signal transduction stage, T/B cells activated protein kinases through complex signaling, leading to the activation and proliferation. In the final stage, activated T/B cells differentiated into subpopulations to perform various biological functions [14]. Research indicated that infections, such as cytomegalovirus and bacteria, could invade endothelial cells or trigger inflammatory cytokines, causing vascular adhesion molecule expression and neutrophil recruitment, leading to vasculitis [15, 16]. This study, based on existing research, investigated both antibodies and immune cell‐specific genes related to infections. It helped us to further understand the relationship between infection immunity and AAV.

Although we did not find a direct relationship between infection‐related immune antibodies and renal damage, we found that the specific gene expression in immune cells caused by infection was related to the occurrence of MPA. We observed that the expression of genes, namely, HLA‐C, MDC1, and MICA, exhibited impacts on MPA. HLA‐C was integral to antigen presentation to CD8+ T cells and played a role in the eradication of infected cells [17]. HLA‐C rs12191877C/T and HLA‐C rs10484554C/T were linked to a decreased risk of rheumatoid arthritis (RA) [18]. Our study further demonstrated that increased expression of HLA‐C in CD8nc T cells was negatively correlated with the risk of MPA, aligning with existing literature. DNA damage checkpoint 1 (MDC1) was located within the major MHC region [19]. The MDC1 protein played a protective role in various tumors and noninfectious diseases, such as noninfectious uveitis, cervical cancer, and ovarian cancer [20–22]. It was plausible that MDC1 in dendritic cells was associated with a reduced risk. Single‐cell transcriptome analysis of PBMCs from MPA demonstrated a significant increase in CD14+ monocytes, while the proportion of classical dendritic cells exhibited a declining trend [23]. These findings underscored the significance of the immune microenvironment and highlighted the importance of gene expression in different immune cells.

MICA was categorized as a nonclassical HLA gene and characterized by a highly polymorphic DNA sequence. Under conditions of cellular stress, MICA played a regulatory role in the immune process through its receptor NKG2D [24]. MICA has been investigated in rheumatic diseases, such as RA, SLE, ankylosing spondylitis (AS), and psoriatic arthritis (PSA) [25]. These studies demonstrated an increase in the MICA allele with rheumatic diseases. We observed that the immune response to infection resulted in elevated MICA expression in NKR cells, which correlated with MPA.

The SCeQTL analysis revealed a correlation between the expression of specific genes in immune cells and MPA, as well as a relationship between these gene expressions and the production of anti‐IgG antibodies against HSV1. Notably, the expression of SMYD3 in CD8s100b T cells exhibited a positive correlation with anti‐IgG levels. In contrast, the expression of CERK in CD8et T cells and NK cells demonstrated negative correlations with anti‐IgG antibodies against HSV1. Previous studies have identified SMYD3 as a member of the methyltransferase SET and MYND domain family, with elevated expression levels being associated with poor prognosis in various cancer types [26]. Recent findings suggest that SMYD3 may serve as a novel regulator of vascular endothelial cell aging by enhancing p21 transcription, thereby contributing to the progression of vascular diseases [27]. The present study indicates that the production of anti‐IgG antibodies against HSV1 is inversely associated with the risk of MPA occurrence. CERK encodes ceramide kinase (CerK), a pivotal enzyme in sphingolipid metabolism [28]. CerK is involved in the regulation of several cellular processes, including cytokine and prostaglandin synthesis, cell migration and invasion, as well as cell proliferation and apoptosis [29–31]. Recent investigations have demonstrated that the knockout of CERK in a murine model of multiple sclerosis confers protection against fundamental processes in CPZ‐treated MS model mice from the adverse effects of oligodendrocyte (OLD) dysfunction and/or loss [32]. Given the current paucity of literature and the limited availability of animal models, further extensive research is imperative to elucidate the roles and clinical significance of SMYD3 and CERK in the context of MPA.

Moreover, this study indicated that DNAmAA exerted a mediating effect between the expression of immune cell‐specific genes and MPA. Recent studies have demonstrated a correlation between DNAmAA and cardiovascular and metabolic diseases [33, 34]. Research on DNAmAA in diabetes has shown an association with microvascular complications [35]. Our study reported that DNAmAA was not only correlated with MPA but also acted as a mediator between immune cell‐specific genes and MPA. This finding was important for understanding the role of DNAmAA in the mechanism of MPA.

This study was subject to several limitations. Despite comprehensive consideration of confounding factors, the absence of cell and animal models made it difficult to draw a causal conclusion. The data from Finns were not representative of the global population, limiting their generalizability to other populations. In future research, we intend to investigate more rigorously to provide clear evidence supporting our results.

## 5. Conclusions

This study demonstrated potential correlations between infection‐related immune responses and MPA. The SCeQTL analysis revealed that HLA‐C expression in CD8nc T cells, MDC1 in dendritic cells, and MICA expression in NKR cells were related to the risk of MPA. Furthermore, mediation analysis indicated that DNAmAA exerted a strong mediation effect between gene expression and MPA.

## Author Contributions

All authors contributed to the study conception and design. Material preparation and data collection were performed by Naidan Zhang, Caixia Ji, Tong Lv, and Xiao Bao. Analysis was performed by Naidan Zhang. The first draft of the manuscript was written by Naidan Zhang. All authors commented on previous versions of the manuscript.

## Funding

This work was supported by the project of the Hospital of Chengdu University of Traditional Chinese Medicine (XJ2023009701), the project of Deyang City science and technology bureau (2024SZY003), and the teaching reform project of Chengdu University of Traditional Chinese Medicine (JGJD202435).

## Disclosure

All authors read and approved the final manuscript.

## Ethics Statement

All summary statistics included in the GWASs and consortia used in this analysis had received approval from the relevant ethical review boards, and participants provided informed consent. This study represents a secondary analysis of publicly available data and does not require additional ethical approval.

## Conflicts of Interest

The authors declare no conflicts of interest.

## Supporting Information

Additional supporting information can be found online in the Supporting Information section.

## Supporting information


**Supporting Information 1** Supporting Table S1: 46 antibody datasets from the U.K. Biobank.

**Supporting Information 2** Supporting Table S2: The characteristics of AAV subtypes.

**Supporting Information 3** Supporting Table S3: The characteristics of 14 types of immune cells.

**Supporting Information 4** Supporting Table S4: Data sources of NET factors.

**Supporting Information 5** Supporting Table S5: Data sources of aging factors.

**Supporting Information 6** Supporting Table S6: Genes associated with MPA.

**Supporting Information 7** Supporting Table S7: The optimal power by pickSoftThreshold.

**Supporting Information 8** Supporting Table S8: SCeQTL showed three genes in immune cells that affect antibodies.

**Supporting Information 9** Supporting Table S9: SCeQTL showed three genes in immune cells that affect MPA.

**Supporting Information 10** Supporting Table S10: Reverse MR analysis of NETs datasets and MPA.

**Supporting Information 11** Supporting Table S11: MPO as a mediator between antibodies/immune cell genes and AAV.

**Supporting Information 12** Supporting Table S12: MR analysis of NETs datasets and MPA.

**Supporting Information 13** Supporting Table S13: Aging‐related phenotypes and MPA.

**Supporting Information 14** Supporting Table S14: Aging as a mediator between infection immunity and MPA.

**Supporting Information 15** STROBE‐MR checklist of recommended items to address in reports of Mendelian randomization studies.

## Data Availability

The data that support the findings of this study are available from the corresponding author upon reasonable request.
