# Supplementary material for: Impact of Infection‐Related Immune Responses on ANCA‐Associated Vasculitis in Finns: A Mendelian Randomization Study
Source: Genet Res (Camb). 2026 Aug 23;2026:3406375. doi: 10.1155/genr/3406375 (PMC13500893; doi:10.1155/genr/3406375)
Supplement: Supplementary file 1 [file GENR-2026-3406375-s001.docx]

**Supplementary Table S3** The characteristics of 14 types of immune cells

| **Cell Type** | **Cell Description** |
| --- | --- |
| CD4et | CD4+KLRB1+ T cell |
| CD4nc | CD4+KLRB1- T cell |
| CD4sox4 | CD4+SOX4+ T cell |
| CD8nc | CD8+LTB+ T cell |
| CD8et | CD8+GNLY+NKG7+ T cell |
| CD8s100b | CD8+S100B+ T cell |
| NK | XCL1- NK |
| NKR | XCL1+ NK |
| Bmen | TCL1A-FCER2- B cell |
| Bin | TCL1A+FCER2+ B cell |
| Plasma | IgJ+ B cell |
| Monoc | Monocyte CD14+ |
| Mononc | Monocyte FCGR3A+ |
| DC | Dendritic cell |

Abbreviation: CD, cluster of differentiation; NK, natural killer cell.
