# Supplementary material for: Impact of Infection‐Related Immune Responses on ANCA‐Associated Vasculitis in Finns: A Mendelian Randomization Study
Source: Genet Res (Camb). 2026 Aug 23;2026:3406375. doi: 10.1155/genr/3406375 (PMC13500893; doi:10.1155/genr/3406375)
Supplement: Supplementary file 2 [file GENR-2026-3406375-s002.docx]

**Supplementary Table S4** Data source of NET factors

| **Exposure** | **Open GWAS ID** | **Sample Size** |
| --- | --- | --- |
| Neutrophil Extracellular Traps (NETs) | ebi-a-GCST90137414 | 657 |
| Interleukin-6 (IL-6) | ebi-a-GCST004446 | 8189 |
| Tumor necrosis factor alpha (TNF-a) | ebi-a-GCST004426 | 3454 |
| Macrophage metalloelastase (NE) | prot-a-1912 | 3301 |
| Neutrophil gelatinase-associated lipocalin (NGAL) | prot-a-1709 | 3301 |
| Myeloperoxidase (MPO) | prot-a-1930 | 3301 |
| Neutrophil-activating peptide 2 | prot-a-2337 | 3301 |
| Myeloperoxidase-DNA complexes (MPO-DNA) | ebi-a-GCST90013658 | 5590 |
