# Supplementary material for: Impact of Infection‐Related Immune Responses on ANCA‐Associated Vasculitis in Finns: A Mendelian Randomization Study
Source: Genet Res (Camb). 2026 Aug 23;2026:3406375. doi: 10.1155/genr/3406375 (PMC13500893; doi:10.1155/genr/3406375)
Supplement: Supplementary file 3 [file GENR-2026-3406375-s003.docx]

**Supplementary Table S5** Data source of aging factors

| **Exposure** | **Open GWAS ID** | **Sample Size** |
| --- | --- | --- |
| DNA methylation GrimAge acceleration | ebi-a-GCST90014288 | 34467 |
| DNA methylation Hannum age acceleration | ebi-a-GCST90014289 | 34449 |
| Intrinsic epigenetic age acceleration | ebi-a-GCST90014290 | 34461 |
| DNA methylation PhenoAge acceleration | ebi-a-GCST90014292 | 34463 |
| Telomere length | ieu-b-4879 | 472174 |
| Frailty index | ebi-a-GCST90020053 | 175226 |
