# Supplementary material for: Impact of Infection‐Related Immune Responses on ANCA‐Associated Vasculitis in Finns: A Mendelian Randomization Study
Source: Genet Res (Camb). 2026 Aug 23;2026:3406375. doi: 10.1155/genr/3406375 (PMC13500893; doi:10.1155/genr/3406375)
Supplement: Supplementary file 4 [file GENR-2026-3406375-s005.docx]

**Supplementary Table S7** The optimal power by ickSoftThreshold

| **Power** | **SFT.R.sq** | **Slope** | **Truncated.R.sq** | **Mean.k** | **Max.k** |
| --- | --- | --- | --- | --- | --- |
| 1 | 0.108 | -0.149 | 0.126 | 1450.00 | 2580.0 |
| 2 | 0.689 | -0.572 | 0.600 | 612.00 | 1600.0 |
| 3 | 0.757 | -0.735 | 0.716 | 323.00 | 1100.0 |
| 4 | 0.780 | -0.835 | 0.788 | 191.00 | 803.0 |
| 5 | 0.810 | -0.902 | 0.840 | 122.00 | 605.0 |
| 6 | 0.823 | -0.966 | 0.868 | 81.50 | 466.0 |
| 7 | 0.840 | -1.000 | 0.889 | 56.60 | 366.0 |
| 8 | 0.853* | -1.030 | 0.906 | 40.50 | 291.0 |
| 9 | 0.876 | -1.050 | 0.923 | 29.70 | 235.0 |
| 10 | 0.887 | -1.060 | 0.933 | 22.20 | 191.0 |
| 12 | 0.909 | -1.060 | 0.946 | 13.10 | 130.0 |
| 14 | 0.943 | -1.050 | 0.965 | 8.15 | 90.4 |
| 16 | 0.936 | -1.090 | 0.969 | 5.29 | 68.5 |
| 18 | 0.911 | -1.140 | 0.951 | 3.56 | 53.1 |
| 20 | 0.888 | -1.180 | 0.936 | 2.47 | 42.0 |
