# Supplementary material for: Impact of Infection‐Related Immune Responses on ANCA‐Associated Vasculitis in Finns: A Mendelian Randomization Study
Source: Genet Res (Camb). 2026 Aug 23;2026:3406375. doi: 10.1155/genr/3406375 (PMC13500893; doi:10.1155/genr/3406375)
Supplement: Supplementary file 5 [file GENR-2026-3406375-s006.docx]

**Supplementary Table S8** SCeQTL showed three genes in immune cells affect antibodies

| **Exposure** | **NSNP** | **Method** | **B** | **Se** | **OR** | **95% lower limit** | **95% upper limit** | ***P* value** |
| --- | --- | --- | --- | --- | --- | --- | --- | --- |
| CERK_cd8et | 4 | Inverse variance weighted | -0.365 | 0.172 | 0.694 | 0.496 | 0.972 | 0.033 |
| CERK_cd8et | 4 | BWMR | -0.392 | - | 0.675 | 0.457 | 0.998 | 0.049 |
| CERK_nk | 5 | Inverse variance weighted | -0.505 | 0.147 | 0.604 | 0.452 | 0.805 | 0.0006 |
| CERK_nk | 5 | BWMR | -0.519 | - | 0.595 | 0.428 | 0.827 | 0.002 |
| SMYD3_cd8s100b | 4 | Inverse variance weighted | 0.506 | 0.191 | 1.659 | 1.141 | 2.413 | 0.008 |
| SMYD3_cd8s100b | 4 | BWMR | 0.521 | - | 1.683 | 1.099 | 2.579 | 0.017 |
