# Supplementary material for: Impact of Infection‐Related Immune Responses on ANCA‐Associated Vasculitis in Finns: A Mendelian Randomization Study
Source: Genet Res (Camb). 2026 Aug 23;2026:3406375. doi: 10.1155/genr/3406375 (PMC13500893; doi:10.1155/genr/3406375)
Supplement: Supplementary file 6 [file GENR-2026-3406375-s007.docx]

**Supplementary Table S9** SCeQTL showed three genes in immune cells affect MPA

| **Exposure** | **NSNP** | **Method** | **B** | **Se** | **OR** | **95% lower limit** | **95% upper limit** | ***P* value** |
| --- | --- | --- | --- | --- | --- | --- | --- | --- |
| HLA-C_cd8nc | 17 | Inverse variance weighted | -0.672 | 0.169 | 0.510 | 0.366 | 0.711 | 7.19E-05 |
| HLA-C_cd8nc | 17 | BWMR | -0.711 | - | 0.491 | 0.348 | 0.692 | 4.98E-05 |
| HLA-C_nk | 14 | Inverse variance weighted | -0.770 | 0.152 | 0.462 | 0.343 | 0.624 | 4.73E-07 |
| HLA-C_nk | 14 | BWMR | -0.783 | - | 0.457 | 0.335 | 0.624 | 8.53E-07 |
| HLA-C_plasma | 3 | Inverse variance weighted | -1.032 | 0.324 | 0.355 | 0.188 | 0.672 | 0.001 |
| HLA-C_plasma | 3 | BWMR | -1.042 | - | 0.352 | 0.180 | 0.689 | 0.002 |
| MDC1_dc | 29 | Inverse variance weighted | -0.460 | 0.123 | 0.631 | 0.495 | 0.804 | 2.02E-04 |
| MDC1_dc | 29 | BWMR | -0.455 | - | 0.634 | 0.500 | 0.805 | 1.82E-04 |
| MICA_nkr | 34 | Inverse variance weighted | 0.456 | 0.117 | 1.578 | 1.252 | 1.987 | 1.06E-04 |
| MICA_nkr | 34 | BWMR | 0.450 | - | 1.568 | 1.227 | 2.004 | 3.20E-04 |
