# Supplementary material for: Impact of Infection‐Related Immune Responses on ANCA‐Associated Vasculitis in Finns: A Mendelian Randomization Study
Source: Genet Res (Camb). 2026 Aug 23;2026:3406375. doi: 10.1155/genr/3406375 (PMC13500893; doi:10.1155/genr/3406375)
Supplement: Supplementary file 7 [file GENR-2026-3406375-s008.docx]

**Supplementary Table S10** Reverse MR analysis of NETs datasets and MPA

| **Exposure** | **Outcome** | **NSNP** | **Beta** | **OR** | ***P* value** |
| --- | --- | --- | --- | --- | --- |
| MPA | NETs | 19 | -0.079 | 0.924 | 0.141 |
| MPA | IL-6 | 15 | -0.003 | 0.997 | 0.790 |
| MPA | TNF-a | 15 | 0.003 | 1.003 | 0.884 |
| MPA | NE | 17 | -0.010 | 0.990 | 0.487 |
| MPA | NGAL | 17 | -0.012 | 0.988 | 0.485 |
| MPA | MPO | 17 | 0.012 | 1.012 | 0.411 |
| MPA | MPO-DNA | 6 | -0.010 | 0.990 | 0.618 |
| MPA | NAP-2 | 17 | -0.015 | 0.986 | 0.426 |

Abbreviation: MR, mendelian randomization; NETs, neutrophil extracellular traps; MPA, microscopic polyarteritis; IL-6, interleukin-6; TNF-a, tumor necrosis factor-alpha; NE, neutrophil elastase; NGAL, neutrophil gelatinase-associated lipocalin; MPO, myeloperoxidase; NAP-2, neutrophil-activating peptide 2.
