# Supplementary material for: Impact of Infection‐Related Immune Responses on ANCA‐Associated Vasculitis in Finns: A Mendelian Randomization Study
Source: Genet Res (Camb). 2026 Aug 23;2026:3406375. doi: 10.1155/genr/3406375 (PMC13500893; doi:10.1155/genr/3406375)
Supplement: Supplementary file 8 [file GENR-2026-3406375-s009.docx]

**Supplementary Table S11** MPO as a mediator between antibodies/gene immune cells and AAV

| **Mediator** | **Exposure** | **Outcome** | **Beta all** | **Beta1** | **Beta2** | **Z** | ***P* value** |
| --- | --- | --- | --- | --- | --- | --- | --- |
| MPO | GCST90006898 | EGPA | -0.499 | 0.136 | -0.316 | -0.954 | 0.340 |
| MPO | GCST90006929 | EGPA | 0.676 | -0.196 | -0.316 | 1.377 | 0.168 |
| MPO | GCST90006924 | MPA | -0.997 | 0.112 | 0.517 | 1.064 | 0.287 |
| MPO | CCHCR1_nkr | EGPA | 0.515 | -0.147 | -0.316 | 1.049 | 0.294 |
| MPO | CCHCR1_plasma | EGPA | 0.471 | -0.175 | -0.316 | 1.247 | 0.212 |
| MPO | DDX39B_plasma | EGPA | 0.768 | -0.191 | -0.316 | 1.343 | 0.179 |
| MPO | HCG27_cd8s100b | EGPA | 0.443 | -0.122 | -0.316 | 0.873 | 0.382 |
| MPO | HLA-B_bin | EGPA | 0.332 | -0.053 | -0.316 | 0.380 | 0.703 |
| MPO | HLA-B_bmem | EGPA | 0.333 | -0.066 | -0.316 | 0.473 | 0.636 |
| MPO | HLA-B_cd8et | EGPA | 0.303 | -0.050 | -0.316 | 0.358 | 0.719 |
| MPO | HLA-B_cd8nc | EGPA | 0.270 | -0.050 | -0.316 | 0.356 | 0.721 |
| MPO | HLA-B_cd8s100b | EGPA | 0.309 | -0.057 | -0.316 | 0.407 | 0.683 |
| MPO | HLA-B_monoc | EGPA | 0.268 | -0.055 | -0.316 | 0.398 | 0.690 |
| MPO | HLA-B_nk | EGPA | 0.307 | -0.044 | -0.316 | 0.315 | 0.752 |
| MPO | HLA-B_plasma | EGPA | 0.540 | -0.073 | -0.316 | 0.523 | 0.600 |
| MPO | MICA_dc | EGPA | -0.614 | 0.172 | -0.316 | -1.223 | 0.221 |
| MPO | MICA_nkr | EGPA | 0.672 | -0.172 | -0.316 | 1.220 | 0.222 |
| MPO | MSH5_cd8et | EGPA | 1.068 | -0.270 | -0.316 | 1.616 | 0.106 |
| MPO | PBX2_cd4sox4 | EGPA | -0.752 | 0.157 | -0.316 | -1.099 | 0.271 |
| MPO | POU5F1_cd8et | EGPA | -0.301 | 0.058 | -0.316 | -0.418 | 0.675 |
| MPO | POU5F1_nk | EGPA | 0.520 | -0.156 | -0.316 | 1.114 | 0.264 |
| MPO | CCHCR1_nkr | MPA | 0.290 | -0.147 | 0.517 | -1.405 | 0.159 |
| MPO | CCHCR1_plasma | MPA | 0.151 | -0.175 | 0.517 | -1.670 | 0.094 |
| MPO | HCG27_bmem | MPA | -0.224 | 0.118 | 0.517 | 1.129 | 0.258 |
| MPO | HLA-B_bin | MPA | 0.206 | -0.053 | 0.517 | -0.508 | 0.610 |
| MPO | HLA-B_bmem | MPA | 0.220 | -0.066 | 0.517 | -0.633 | 0.526 |
| MPO | HLA-B_plasma | MPA | 0.492 | -0.073 | 0.517 | -0.700 | 0.483 |
| MPO | MICA_nkr | MPA | 0.456 | -0.172 | 0.517 | -1.637 | 0.101 |
| MPO | MSH5_cd4et | MPA | -0.277 | -0.111 | 0.517 | -1.063 | 0.287 |
| MPO | POU5F1_cd8et | MPA | -0.248 | 0.058 | 0.517 | 0.559 | 0.575 |
| MPO | POU5F1_nk | MPA | 0.268 | -0.156 | 0.517 | -1.492 | 0.135 |
| MPO | TCF19_mononc | MPA | -0.489 | -0.086 | 0.517 | -0.825 | 0.409 |
