# Supplementary material for: Impact of Infection‐Related Immune Responses on ANCA‐Associated Vasculitis in Finns: A Mendelian Randomization Study
Source: Genet Res (Camb). 2026 Aug 23;2026:3406375. doi: 10.1155/genr/3406375 (PMC13500893; doi:10.1155/genr/3406375)
Supplement: Supplementary file 9 [file GENR-2026-3406375-s010.docx]

**Supplementary Table S12** MR analysis of NETs datasets and MPA

| **Exposure** | **NSNP** | **Beta** | **OR** | **95% CI** | ***P* value** |
| --- | --- | --- | --- | --- | --- |
| Frailty index | 237 | 2.821 | 16.792 | 5.723-49.269 | 2.79E-07 |
| DNA methylation GrimAge acceleration | 38 | 0.077 | 1.080 | 0.872-1.339 | 0.477 |
| DNA methylation Hannum age acceleration | 103 | 0.257 | 1.293 | 1.137-1.472 | 9.14E-05 |
| Intrinsic epigenetic age acceleration | 119 | -0.016 | 0.984 | 0.890-1.086 | 0.750 |
| DNA methylation PhenoAge acceleration | 57 | 0.012 | 1.012 | 0.894-1.145 | 0.846 |
| Telomere length | 1282 | -0.186 | 0.829 | 0.550-1.250 | 0.372 |

Abbreviation: MR, mendelian randomization; NETs, neutrophil extracellular traps; MPA, microscopic polyarteritis.
