# Supplementary material for: Impact of Infection‐Related Immune Responses on ANCA‐Associated Vasculitis in Finns: A Mendelian Randomization Study
Source: Genet Res (Camb). 2026 Aug 23;2026:3406375. doi: 10.1155/genr/3406375 (PMC13500893; doi:10.1155/genr/3406375)
Supplement: Supplementary file 10 [file GENR-2026-3406375-s011.docx]

**Supplementary Table S2** The characteristics of AAV subtypes

| **AAV subtypes** | **Number of cases** | **Number of controls** | **Path https** |
| --- | --- | --- | --- |
| Microscopic polyarteritis | 166 | 451441 | https://storage.googleapis.com/finngen-public-data-r11/summary_stats/finngen_R11_M13_MICROPOLYANG.gz |
| Granulomatosis with polyangiitis | 486 | 439424 | https://storage.googleapis.com/finngen-public-data-r11/summary_stats/finngen_R11_M13_WEGENER.gz |
| Eosinophilic granulomatosis with polyangiitis | 118 | 439424 | https://storage.googleapis.com/finngen-public-data-r11/summary_stats/finngen_R11_M13_CHURGSTRAUSS.gz |
