# Supplementary material for: Impact of Infection‐Related Immune Responses on ANCA‐Associated Vasculitis in Finns: A Mendelian Randomization Study
Source: Genet Res (Camb). 2026 Aug 23;2026:3406375. doi: 10.1155/genr/3406375 (PMC13500893; doi:10.1155/genr/3406375)
Supplement: Supplementary file 11 [file GENR-2026-3406375-s012.docx]

**Supplementary Table S1** 46 antibody datasets from the UK Biobank

| **Infectious Agents** | **Antigen Targets** | **Open GWAS ID** |
| --- | --- | --- |
| Human herpes virus 7 | U14 | GCST90006909 |
|  | IgG | GCST90006908 |
| helicobacter pylori | IgG | GCST90006910 |
|  | CagA | GCST90006911 |
|  | Catalase | GCST90006912 |
|  | GroEL | GCST90006913 |
|  | OMP | GCST90006914 |
|  | UREA | GCST90006915 |
|  | VacA | GCST90006916 |
| herpes simplex virus 1 | IgG | GCST90006917 |
|  | mgG-1 | GCST90006918 |
| herpes simplex virus 2 | IgG | GCST90006919 |
|  | mgG-1 | GCST90006920 |
| Polyomavirus 2 | IgG | GCST90006921 |
|  | JC VP1 | GCST90006922 |
| Merkel cell polyomavirus | IgG | GCST90006923 |
|  | VP1 | GCST90006924 |
| Toxoplasma gondii | IgG | GCST90006925 |
|  | p22 | GCST90006926 |
|  | sag1 | GCST90006927 |
| Varicella zoster virus | IgG | GCST90006928 |
|  | glycoproteins E and I | GCST90006929 |
| BK polyomavirus | VP1 | GCST90006885 |
|  | IgG | GCST90006884 |
| Chlamydia trachomatis | IgG | GCST90006886 |
|  | momp A | GCST90006887 |
|  | momp D | GCST90006888 |
|  | pGP3 | GCST90006889 |
|  | PorB | GCST90006890 |
|  | tarp-D F1 | GCST90006891 |
|  | tarp-D F2 | GCST90006892 |
| Cytomegalovirus | IgG | GCST90006893 |
|  | pp28 | GCST90006894 |
|  | pp52 | GCST90006895 |
|  | pp150 | GCST90006896 |
| Epstein-Barr virus | IgG | GCST90006897 |
|  | EA-D | GCST90006898 |
|  | EBNA-1 | GCST90006899 |
|  | VCA p18 | GCST90006900 |
|  | ZEBRA | GCST90006901 |
| Human herpes virus 6 | IgG | GCST90006902 |
|  | Ant E1A IgG seropositivity | GCST90006903 |
|  | IE1A | GCST90006904 |
|  | IE1B | GCST90006906 |
|  | Ant IE1B IgG seropositivity | GCST90006905 |
|  | p101k | GCST90006907 |
