# Supplementary material for: Impact of Infection‐Related Immune Responses on ANCA‐Associated Vasculitis in Finns: A Mendelian Randomization Study
Source: Genet Res (Camb). 2026 Aug 23;2026:3406375. doi: 10.1155/genr/3406375 (PMC13500893; doi:10.1155/genr/3406375)
Supplement: Supplementary file 12 [file GENR-2026-3406375-s013.docx]

**Supplementary Table S14** Aging as a mediator between infection immunity and MPA

| **Mediator** | **Exposure** | **Outcome** | **Beta_all** | **Beta1** | **Beta2** | **Z** | ***P* value** | **Mediation effect (%)** |
| --- | --- | --- | --- | --- | --- | --- | --- | --- |
| DNAmAA | GCST90006901 | MPA | 0.545 | -0.327 | 0.257 | -2.695 | 0.007* | - |
| DNAmAA | GCST90006924 | MPA | -0.997 | 0.197 | 0.257 | 2.121 | 0.033* | - |
| DNAmAA | HLA-C_cd8nc | MPA | -0.672 | -0.462 | 0.257 | -2.921 | 0.003 | -0.553 (82.2%) |
| DNAmAA | HLA-C_nk | MPA | -0.770 | -0.421 | 0.257 | -2.858 | 0.004 | -0.661 (85.9%) |
| DNAmAA | MDC1_dc | MPA | -0.460 | -0.331 | 0.257 | -3.310 | 0.001 | -0.374 (81.5%) |
| DNAmAA | MICA_nkr | MPA | 0.456 | 0.493 | 0.257 | 3.702 | <0.001 | 0.329 (72.1%) |

Abbreviation: AAV, Anti-neutrophil cytoplasmic antibody associated vasculitis; TL, telomere length; DNAmAA, DNA methylation Hannum age acceleration; EGPA, eosinophilic granulomatosis with polyangiitis; MPA, microscopic polyarteritis.
