# Supplementary material for: Impact of Infection‐Related Immune Responses on ANCA‐Associated Vasculitis in Finns: A Mendelian Randomization Study
Source: Genet Res (Camb). 2026 Aug 23;2026:3406375. doi: 10.1155/genr/3406375 (PMC13500893; doi:10.1155/genr/3406375)
Supplement: Supplementary file 14 [file GENR-2026-3406375-s015.docx]

**Supplementary Table S13** Aging-related phenotypes and MPA

| **Exposure** | **Heterogeneity** | | **Pleiotropy** | | |
| --- | --- | --- | --- | --- | --- |
|  | **Q** | ***P*** _Q_ **value** | **Egger intercept** | **SE** | ***P* value** |
| DNA methylation GrimAge acceleration | 85.76 | 0.876 | 0.009 | 0.036 | 0.801 |
| Frailty index | 397.31 | <0.001* | -0.151 | 0.038 | <0.001* |

Abbreviation: DNAmAA, DNA methylation Hannum age acceleration; FI, frailty index; MPA, microscopic polyarteritis.
